# Systemic Health System Measurement Framework: An Approach Based on the Unified Care Model for Whole Systems Transformation

**DOI:** 10.3390/healthcare14091141

**Published:** 2026-04-24

**Authors:** Ther Lim, Yun Hu, Ada Wah Yean Lee, Jit Kai Tan, Qi Yin Ngoi, Naiying Liu, Audrey Cai Ling Tay, Justin Guang Jie Lee, Yeuk Fan Ng

**Affiliations:** 1 Khoo Teck Puat Hospital & Yishun Community Hospital, NHG Health, 90 Yishun Central, Singapore 768828, Singapore; lim.ther@nhghealth.com.sg (T.L.); hu.yun@nhghealth.com.sg (Y.H.); justin.lee.gj@nhghealth.com.sg (J.G.J.L.); 2 Saw Swee Hock School of Public Health, National University of Singapore, 12 Science Drive 2, Singapore 117549, Singapore; 3 Health Services Research & Population Health Program, Duke-NUS Medical School, 8 College Road, Singapore 169857, Singapore

**Keywords:** population health measurement, integrated care, health systems reform, quality indicators, systemic design, unified care model, population segmentation

## Abstract

**Highlights:**

**What are the main findings?**

- The Systemic Health System Measurement Framework (SHSMF) organises indicators around needs-based population segments and cascades them across macro–meso–micro levels, integrating lifelong and episodic care within a Unified Care Model.
- Implemented at Yishun Health via governance and performance dashboards from 2022 to 2024, SHSMF dashboards (covering ~208,000 residents across seven lifelong care segments and ~230,000 inpatient cases across six episodic care segments) revealed systemic patterns and higher-leverage intervention points not readily apparent in conventional dashboards. Based on descriptive segment-level analyses, psychosocial complexity was consistently associated with disproportionate costs despite lower medical acuity.

**What are the implications of the main findings?**

- Whole-system transformation efforts require measurement systems that transcend disease and provider silos. Systemic Health System Measurement Framework (SHSMF) architectures can strengthen accountability by linking population needs, integrated care processes, and outcomes across system levels, supporting governance focused on system performance rather than service-line optimisation alone.
- Addressing psychosocial complexity through appropriately supported, integrated models of care may yield outsized value (cost containment and outcome gains) compared to medical acuity-focused approaches alone. The framework and dashboard approach is transferable for health systems pursuing value-based whole-systems population health management.

**Abstract:**

**Background/Objectives:** Health systems globally are transforming toward population-based, person-centred care, yet measurement systems frequently remain anchored in provider-centric or disease-specific frameworks. This paper presents the Systemic Health System Measurement Framework (SHSMF), a population health systems measurement architecture that completes a conceptual systemic health systems design and transformation trilogy with the Unified Care Model (UCM) and Systemic Health System Population Segmentation Model, addressing how health systems can measure whether systems integration is succeeding. **Methods:** This study employs a conceptual framework development and implementation case study design approach, with the Systemic Health System Measurement Framework (SHSMF) developed using the Health System Transformation Playbook (HSTP) methodology. The framework organises measurement around needs-based population segments, integrates Lifelong Care and Episodic Care measurement within a unified architecture, and cascades indicators across macrosystem, mesosystem and microsystem levels. Implementation was demonstrated through the development of performance and governance dashboards development at Yishun Health, a regional population health system serving approximately 320,000 residents in Singapore (2022–2024). **Results:** Descriptive analytics from the Lifelong Care Dashboard (207,980 residents across seven segments) and the Episodic Care Dashboard (230,365 inpatient cases across six segments) revealed systemic patterns not readily apparent through conventional approaches. Psychosocial complexity was consistently associated with disproportionate cost trends across both dashboards despite lower medical acuity. Quality indicator performance across psychosocially complex segments was not proportionally worse, yet these segments bore disproportionate costs, a pattern consistent with the view that segment-specific care redesign addressing psychosocial needs may be associated with both an improvement in outcomes and cost efficiencies. **Conclusions:** The Systemic Health System Measurement Framework (SHSMF) demonstrates that a measurement architecture explicitly designed around systemic needs-based population segments improves systemic health systems accountability and provides governance opportunities that conventional approaches may not achieve. The framework and its dashboard implementation offer a transferable methodology for health systems globally seeking to implement a whole-systems measurement architecture for value-based population health management.

## 1. Introduction

### 1.1. Health System Transformation and the Measurement Gap

Health systems worldwide are undergoing a fundamental transformation driven by ageing populations, rising chronic disease burden, and workforce constraints [1]. Large-scale transformation through coordinated, system-wide change is increasingly recognised as necessary [2], requiring a shift from episodic, provider-centred healthcare focused on treating disease within institutional boundaries, toward continuous, person-centred population health systems that optimise health and wellbeing across entire populations throughout the life-course [3,4]. Despite these reforms, measurement systems have not evolved at the same pace, creating a structural misalignment between transformation ambitions and performance governance [5,6].

Population segmentation approaches offer significant potential by stratifying populations according to their needs [7,8], yet measurement systems have not evolved correspondingly to provide segment-level accountability. Consequently, health system stewards lack coherent visibility into whole-system performance relative to specific provider organisations and disease-based programmes, and accountability for population-level outcomes remains obscured. Emerging approaches to population health measurement have begun to address these gaps [9,10], yet systematic guidance on integrating such approaches within comprehensive measurement architectures remains limited.

### 1.2. Existing Measurement Frameworks and Their Limitations

Performance measurement has long been recognised as essential for health systems improvement, guiding resource allocation, accountability mechanisms, and quality enhancement [5]. Many frameworks have been developed to assess health system performance at various levels, each offering distinct contributions while revealing limitations when applied to the challenge of measuring whole-system transformation toward population-based, person-centred care. Table 1 describes examples of common approaches and frameworks, their measurement focus, and the gaps they leave unaddressed for whole-systems enablement.

As Table 1 exemplifies, foundational frameworks such as Donabedian’s structure-process-outcome model [11] and disease-specific measurement initiatives such as those of Porter’s value-based healthcare framework [12,13] and the International Consortium for Health Outcomes Measurement (ICHOM)’s standardised outcome sets [14] and clinical registries [15] have proven valuable for assessing the outcomes and costs of specific clinical services and institutional performance of integrated practice units through their emphasis on condition-specific measurement cycles. However, these frameworks were not designed to address the challenge of measuring the performance of whole-population health systems with heterogeneous needs across multiple system levels. Other contemporary frameworks have attempted more comprehensive approaches: the World Health Organization’s Health System Performance Assessment framework [18] provides system-level conceptual guidance but is primarily oriented toward national-level assessment rather than operational governance within regional health systems. The OECD Health Care Quality Indicators framework [16] developed standardised indicators for international benchmarking but organises them by quality dimensions rather than population segments, while Nelson et al.’s clinical microsystems framework [17] established the principle of nested system levels for quality improvement but does not extend to population-level measurement architecture. The Triple and Quadruple Aim frameworks [19,20] have been widely adopted as a guiding vision but do not sufficiently specify a measurement architecture for operationalising their goal domains within complex, multi-level health systems; and the Learning Health System concept [21] provides a compelling vision for continuous improvement but offers less guidance on the specific measurement architecture needed across population segments and system levels simultaneously.

What emerges from these examples is that each of these foundational frameworks makes important contributions within its intended scope, yet limited guidance exists on designing measurement systems to enable whole-health-system transformation toward population-based, person-centred care [5,22]. Similarly, systematic reviews of population segmentation tools indicate that most segmentation approaches rely on disease categories or healthcare utilisation patterns rather than biopsychosocial needs [23], limiting the extent to which measurement systems can be structured around person-centred care principles. Guidance on integrating the measurement of continuous, longitudinal health maintenance with the measurement of discrete acute care episodes in ways that reflect their nested relationship also remains limited. The Systemic Health System Measurement Framework (SHSMF) is proposed therefore as a response to these unaddressed measurement challenges.

### 1.3. Singapore Context and Conceptual Foundations

In Singapore, the Ministry of Health articulated three fundamental policy shifts for health system sustainability—the “Three Beyonds”—namely, beyond healthcare to health, beyond hospital to community, and beyond quality to value [24]. To operationalise these shifts, Singapore’s healthcare system reorganised into Regional Health Systems, each responsible for the health and healthcare of defined geographic populations [25]. The subsequent Healthier SG initiative further strengthens primary care, with residents enrolling with a regular family doctor responsible for preventive care and holistic health planning [26]. These policy developments create the imperative for systemically designed measurement systems that enable the stewardship of population health outcomes and value, not merely institutional performance metrics.

In 2017, the National Healthcare Group reorganised Khoo Teck Puat Hospital (a 795-bed acute hospital), Yishun Community Hospital (a 428-bed sub-acute, rehabilitative, and palliative care hospital), and the Admiralty Medical Centre (an ambulatory care centre) into Yishun Health as an integrated care organisation serving approximately 320,000 residents within the Yishun Zone Regional Population Health System in Northern Singapore [25]. This reorganisation expanded Yishun Health’s purpose beyond hospital-based healthcare provision to a collaborative stewardship of population health in partnership with primary care providers, including polyclinics (government-subsidised primary care clinics), community health agencies, and social service organisations.

The Unified Care Model (UCM) and Systemic Health System Population Segmentation Model developed at Yishun Health in Singapore provided the conceptual foundations for addressing the interventional needs for the expanded purpose of Yishun Health [25,27], as well as for addressing these aforementioned performance measurement gaps. For example, the UCM articulates person-centred care architecture distinguishing Lifelong Care, defined as the continuous experience of health across the life-course, from the Episodic Care events nested within it. The segmentation model stratifies residents into mutually exclusive groups based on biopsychosocial needs, enabling differentiated care design, and, accordingly, the more systematic measurement of population health system outcomes and performance accountability.

### 1.4. Study Aims

This paper addresses the gap between transformation aspirations and measurement capabilities by presenting the Systemic Health System Measurement Framework (SHSMF). The SHSMF is a systematic population health system outcomes measurement approach anchored on the Unified Care Model [25] and operationalised through the Systemic Health System Population Segmentation Model [27]. This paper aims to: (1) articulate the conceptual foundations and design principles of the SHSMF; (2) describe the framework’s operationalisation through Lifelong Care and Episodic Care measurement architectures cascading across the macrosystem, mesosystem, and microsystem levels; (3) demonstrate the framework’s application through a case study of dashboard implementation in Yishun Health’s regional population health system (2022–2024); and (4) discuss how the SHSMF completes a coherent conceptual systemic health systems design and transformation trilogy comprising systemic population segmentation, segment-specific integrated care interventions, and systemic measurement, providing health systems with a replicable approach to implement a whole-systems measurement architecture for value-based population health management.

## 2. Methods and Materials

### 2.1. Study Design and Validation

This study employs a conceptual framework development and implementation case study design approach, with the Systemic Health System Measurement Framework (SHSMF) developed through an iterative process of stakeholder engagement, international benchmarking, and systemic design, guided by the Health System Transformation Playbook (HSTP) methodology [25]. The framework was anchored on the Unified Care Model (UCM) [25] and the Systemic Health System Population Segmentation Model [27] to enable a coherent, whole-health-systems value-based development approach. Implementation is demonstrated through the deployment of operational performance and governance dashboards at Yishun Health, a regional population health system in Singapore, from 2022 to 2024.

### 2.2. Population Segmentation and Validated Foundations

The SHSMF organises measurement around the population segments defined by the Systemic Health System Population Segmentation Model [27], which stratifies a health system’s resident population into mutually exclusive and collectively exhaustive segments based on biopsychosocial needs, specifically, the Lifelong Care Segmentation Model classifies residents into seven segments (LS1–LS7) using criteria encompassing the stage of chronic disease progression, the presence of mental health issues and social issues. The Needs-Based Sub-Segmentation (NBSS) Model [27] further stratifies residents with chronic diseases (LS3–LS6) into ten sub-segments along two dimensions: disease stage (early disease, denoted “A”, versus advanced disease, denoted “B”) and level of complexity. Sub-segments range from residents with a single chronic disease only (A1, B1) through those with multiple chronic diseases (A2, B2), multiple chronic diseases with mental health issues (A3, B3), multiple chronic diseases with social issues (A4, B4), to those with multiple chronic diseases combined with both mental health and social issues (A5, B5). The Episodic Care Segmentation Model classifies emergency inpatient admissions into six segments (ES1–ES6) by layering acute care acuity as quantified by Patient Acuity Category Scale (PACS) values over the resident’s lifelong care segment. Hu et al. [27] evaluated the Systemic Health System Population Segmentation Model using healthcare utilisation, cost, and readmission outcome data. Chi-square tests, one-way ANOVA, and linear regression analyses demonstrated that all segments differed significantly across key outcome measures (*p* < 0.001). The SHSMF, in turn, builds upon these validated segments, to demonstrate what becomes visible when measurement is systematically organised around them.

### 2.3. Framework Conceptual Foundations and Design Principles

The Systemic Health System Measurement Framework (SHSMF) is anchored on four core principles that distinguish it from conventional health system performance measurement approaches. These principles emerged from the parallel development of the Unified Care Model (UCM) and the Systemic Health System Population Segmentation Model, reflecting the interdependent nature of care model design, population stratification, and measurement architecture in enabling coherent whole-health-system transformation.

The first foundational principle is that measurement should be organised around needs-based population segments rather than provider organisational structures or disease categories alone. Traditional performance measurement systems typically organise indicators by clinical department, disease programme, or institutional provider, reflecting the supply-side structure of healthcare delivery. By contrast, the SHSMF organises indicators around the seven Lifelong Care segments (LS1–LS7) and six Episodic Care segments (ES1–ES6) defined by the systemic population segmentation model [27], enabling performance comparison across populations with systematically different biopsychosocial needs and supporting differential resource allocation aligned with the UCM’s person-centred design principles.

**Figure 1:**
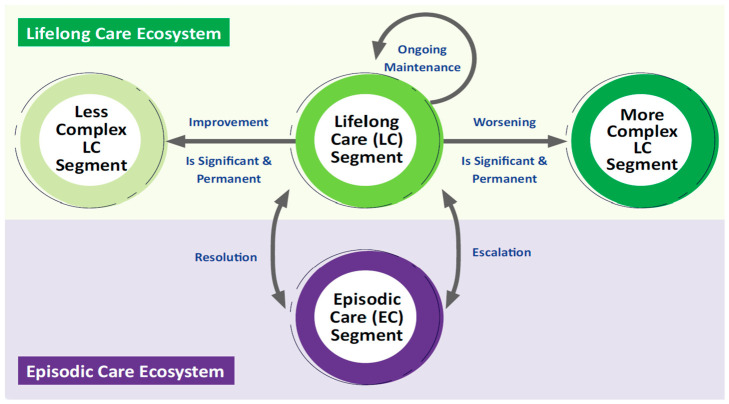
Unified Care Model: flow between Lifelong Care and Episodic Care segments.

The second principle is the integration of Lifelong Care and Episodic Care measurement within a unified framework. The UCM conceptualises Lifelong Care as the continuous, longitudinal experience of health across a resident’s life course, within which discrete Episodic Care events are nested [25]. Figure 1 illustrates this relationship, showing how residents move within and between Lifelong Care segments while acute events trigger escalation to Episodic Care.

The SHSMF reflects this nested relationship by linking episodic care measurement to residents’ Lifelong Care segment membership, enabling analysis of how Lifelong Care outcome statuses, events and behaviours influence Episodic Care outcome statuses and care trajectories, and how Episodic Care events and behaviours affect Lifelong Care outcome statuses and care trajectories.

The third principle concerns indicator organisation within each measurement domain. For Lifelong Care, indicators are organised using the Quadruple Aim framework at population segment level: population health outcomes, residents’ experience, costs, and care team wellbeing [19,20]. For Episodic Care, indicators are organised using the Patient Value Compass framework adapted to population segments, measuring clinical outcomes, functional status, patient experience, and episode costs [28]. This framework-driven organisation ensures indicator selection serves strategic transformation purposes rather than merely reflecting data availability.

The fourth principle is that measurement should enable accountability assignment and continuous improvement, not merely retrospective performance reporting. This requires measurement systems providing timely visibility into segment-specific performance and generating actionable feedback for improvement. The SHSMF was designed explicitly to serve internal transformation purposes, with external reporting as a secondary consideration.

### 2.4. Cascade Measurement Architecture

**Figure 2:**
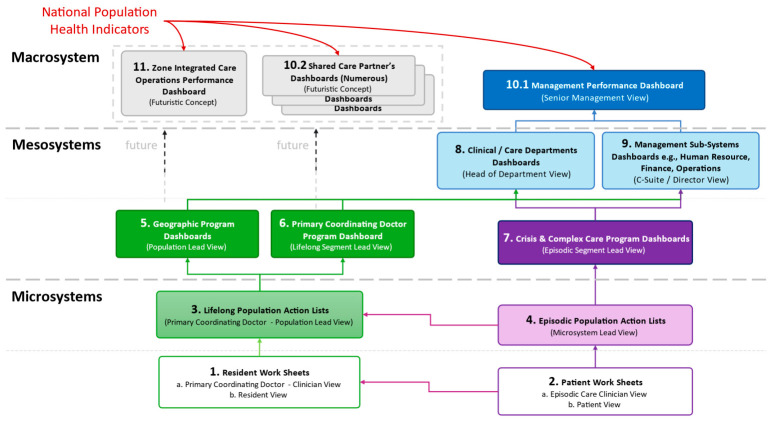
SHSMF conceptual dashboard architecture across macrosystem, mesosystem, and microsystem levels.

The SHSMF employs a conceptual whole-population health system cascade architecture organising measurement across three subsystem levels, building upon Nelson et al.’s concept of nesting clinical microsystems within mesosystems and macrosystems [17], and reflecting a hierarchical structure for population health system governance (Figure 2). Nelson et al.’s clinical microsystems framework [17] established the principle that health systems are composed of nested levels where frontline microsystems are embedded within mesosystems and macrosystems, and the OECD Health Care Quality Indicators framework [16] developed standardised quality indicators for international benchmarking organised by dimensions of care and patient needs. However, neither framework organises measurement around needs-based population segments or integrates longitudinal and episodic care within a unified architecture. The SHSMF cascade architecture builds on this multi-level principle but reorients it: indicators are organised around population segments at each system level, enabling cascaded governance based on population needs within an integrated regional health system.

At the macrosystem level, the Management Performance Dashboard provides whole-system visibility to senior leadership, displaying aggregate population health outcomes, experience, and cost indicators across all segments. The architecture also conceptualises future extensions including Zone Integrated Care Organisation and Shared Care Partner Dashboards as integrated care networks mature. National population health indicators from the Ministry of Health can flow into the macrosystem dashboard, enabling comparison with national benchmarks while maintaining segment-based organisation for internal accountability.

At the mesosystem level, programme-specific dashboards provide visibility to programme and clinical leaders. For Lifelong Care, Primary Care and Community Programme Dashboards enable monitoring of segment-specific and geographic performance. For Episodic Care, Crisis and Complex Care Programme Dashboards enable flow leads to monitor episodic care performance. Clinical Department and Management Sub-Systems Dashboards provide departmental and functional visibility.

At the microsystem level, care team and individual-level tools were conceptualised to enable frontline accountability, including Resident Worksheets, Population Action Lists, and patient views that are potentially accessible through national health IT platforms.

Critically, the Lifelong Care and Episodic Care measurement streams are integrated within this unified architecture rather than operating as separate systems. Each resident is assigned to a Lifelong Care segment based on their biopsychosocial needs, and when that resident experiences an acute event, their episode is assigned to an Episodic Care segment while retaining linkage to their Lifelong Care segment. This enables analysis of how Lifelong Care segment membership influences episodic outcomes (for example, whether psychosocially complex segments experience longer lengths of stay or higher readmission rates) and how episodic events affect Lifelong Care trajectories, such as functional decline following hospitalisation. This bidirectional linkage operationalises the UCM principle that all Episodic Care plans occur within the context of residents’ ongoing lifelong One Care Plans.

### 2.5. Development Methodology

Our approach draws on elements of both design-based research and implementation science: the SHSMF was iteratively developed through cycles of stakeholder engagement, prototyping and refinement consistent with design-based research, while its deployment through operational dashboards within an active health system represents an implementation case study. Specifically, the UCM [25] and Systemic Health System Population Segmentation Model [27] provided the conceptual and operational foundations for SHSMF development, which started as early as 2014. The segmentation model’s validation demonstrated that segments were meaningfully distinct in terms of healthcare utilisation, costs, and outcomes, with psychosocial complexity emerging as a key determinant of poor outcomes across both lifelong and episodic care contexts [27]. Development of the SHSMF was then initiated in 2017 to translate these validated segments into ongoing systemic measurement visibility for whole-population health system performance management and governance.

This parallel development and systemic design approach was intentional: the three components are fundamentally interdependent, with each informing and constraining the others. The UCM’s design principles shaped indicator domains; the segmentation model defined units of analysis; and measurement requirements influenced refinements to care model and service design specifications.

The process of systemic design and development was guided by the Health System Transformation Playbook (HSTP) methodology [25]. The HSTP is an integrated design thinking, systems thinking, and complexity thinking enabled methodology for health system transformation [29,30] that uses a three-step iterative approach: storytelling gathers stakeholder input to identify change ideas; model building co-creates future-state models; and pathfinding identifies interventions and prioritises actions based on organisational context.

For SHSMF development, numerous hospital staff and regional health system senior leaders provided rich narratives about existing measurement challenges such as too few/too many indicators, data fragmentation across providers, unclear accountability for population outcomes, inadequate capture of psychosocial determinants, and delayed data availability. The Corporate Development team at Yishun Health engaged in model building by integrating “storytelling” insights with an understanding of how international health systems, such as the Canterbury District Health Board in New Zealand, NHS England, and Alberta Health Services in Canada, have customised common indicator frameworks (Table 1) into their measurement systems. The team subsequently adapted these relevant principles and practices to the Singapore context using the UCM lens to define the SHSMF and the cascade architecture. Pathfinding then prioritised indicator development and dashboard implementation based on internal stakeholder agreement and data availability.

A critical tactical decision was creating dashboard mock-ups before securing data access. Mock-ups enabled a demonstration of the value proposition to stakeholders controlling data and resources, transforming abstract measurement discussions into concrete visualisations. When engaging the polyclinic institution for data sharing, we created mock-ups using hospital data for segments typically managed by polyclinics, generating enthusiasm for collaboration before formal approval was secured.

Stakeholder engagement employed differential strategies. Senior leadership were provided regular updates on a three-to-six-month cadence to sustain interest and momentum. Clinical leadership engagement surfaced tension between clarity and the differentiation of accountability roles for future-oriented population health system segment performance management indicators versus the more conventional and current clinical and operational metrics for disease-based and institution-based services and profession-centric departments; we designed dashboards to serve both purposes concurrently whenever possible and presented all available dashboards in the spirit of continuous improvement and innovation rather than just for performance management. Notably, the analyses made possible by these dashboards are descriptive in nature; they are intended to illustrate the types of systemic patterns that the SHSMF architecture makes visible for performance management, governance, and quality improvement decision-making, rather than to test specific statistical hypotheses or establish causal relationships. Frontline engagement proceeded based on demonstrated individual department and senior clinician leader readiness and interest, with early adopters providing feedback that refined functionality before broader deployment.

### 2.6. Dashboard Development and Implementation

SHSMF development and implementation proceeded through several stages. Both Lifelong Care and Episodic Care dashboards were conceptualised simultaneously during 2017–2021, but implementation sequencing reflected strategic and tactical decisions. Following indicator development from April 2021 to June 2022, the Lifelong Care Dashboard began development and launched in 2022, reflecting the strategic primacy of population health measurement and enabling coalition-building with our primary care polyclinic partner to launch the Primary Coordinating Doctor (PCD) programme in the hospital’s Integrated Medical Clinic and the polyclinic’s Primary Care Teamlet programme. Both programmes were designed to champion lifelong “One Care Plans” (OCP), starting with patients known to us and gradually expanding beyond this group. The Episodic Care Dashboard followed in 2023, primarily for the acute and community hospital, completing the Unified Care Model systemic measurement architecture. Both dashboards remain operational to date with minor and progressive refinements based on stakeholder feedback.

### 2.7. Data Integration, Data Quality, and Indicator Selection

The SHSMF employs an integrated data architecture synthesising information from multiple sources. For initial implementation, we adopted a pragmatic approach focused on available data rather than comprehensive integration. Primary sources included the hospital’s Central Data Repository, which contains administrative and clinical data (demographic, diagnostic, utilisation, and cost) drawn from the electronic medical record system, and selected polyclinic data obtained through negotiated data sharing.

For the dashboard implementation, the segment and sub-segment assignment described in Section 2.2 was applied to the extracted data. Extract–transform–load processes linked and aggregated data to the resident and episode levels, with segmentation logic applied and outcomes calculated for each segment. Data were linked to care teams to enable accountability assignment. Refresh cycles operated quarterly for episodic care and six-monthly for lifelong care dashboards. Data validation was performed by data analysts who verified the accuracy and completeness of dashboard outputs against the source clinical data from the electronic medical record system. Dashboards are published and shared on the Oracle Analytics Server (OAS).

Indicator development occurred from April 2021 to June 2022, involving a literature review, stakeholder engagement, and iterative refinement. For episodic care, indicators were organised into four priority tiers: Tier 1, meeting both regulatory reporting and patient-centred needs; Tier 2, meeting regulatory needs; Tier 3, meeting patient-centred needs; and Tier 4, meeting process improvement needs. For lifelong care, indicators were organised using the Quadruple Aim framework [19,20].

A critical design decision was that dashboards would serve dual purposes: displaying future-oriented population health indicators alongside current operational metrics. This increased technical and stakeholder usage complexity but proved essential for securing support during stakeholder engagements in the transition period.

## 3. Results: Case Study of Systemic Health System Measurement Framework Implementation at Yishun Health

### 3.1. From Framework to Implementation

The SHSMF provides a comprehensive conceptual whole-systems measurement architecture for population health system measurement and transformation. Guided by this framework, Yishun Health initiated an iterative process of dashboard development, progressively building components and content structures to approximate the measurement needs prescribed by the SHSMF. The dashboards presented in this section represent the current state of this ongoing implementation rather than a complete realisation of the framework’s full scope.

This phased approach reflects the practical realities of health system transformation. Initial development prioritised outcome indicators that could demonstrate the value of segment-level measurement to stakeholders and secure ongoing support for continued development. Process and output indicators, while more comprehensively specified in the indicator framework and although they continue to be refined (Appendix A and Appendix B), remain in various stages of development. In many cases, the underlying data already exists within separate clinical quality, safety, operations, and financial dashboards across the organisation. Progressive planned enhancement will involve ingesting and consolidating these data and indicators into the Lifelong Care and Episodic Care or other dashboards guided by the SHSMF, enabling progressively more process and output measures to be visualised systemically alongside outcomes and within the context of population segment performance. While some absolute cost values in the dashboard figures have been masked in accordance with institutional data governance policies, the relative and proportional relationships across segments are still visible, enabling cost concentration patterns and segment-level variations to be inferred.

Accordingly, the case study that follows illustrates both the framework’s practical application and the realities of incremental whole-population health system measurement development, where data quality and indicator coverage mature progressively over successive implementation cycles.

### 3.2. Lifelong Care Dashboard

The Lifelong Care Dashboard is designed to provide continuous monitoring of health and outcomes for Yishun Zone residents in Singapore’s northern region. It offers insight into the care plan intervention process and its outcomes, stratified across population segments using the Systemic Health System Population Segmentation Model.

The dashboard is designed exclusively for Yishun Zone residents, with filtering by population sub-segment, age group, and enrolment status in key care programmes (Primary Care Teamlet and Integrated Medical Clinic).

Indicators are organised into two complementary domains (Appendix A). Outcome indicators follow the Quadruple Aim framework: population health (protective health factors, chronic disease prevalence, frailty, quality of life, mental wellbeing), experience (residents’ experience of health), costs (health services utilisation and cost per capita), and care team wellbeing. Process and output indicators are structured around the One Care Plan journey in three phases: (LA) Population Segmentation, including Primary Coordinating Doctor assignment; (LB) Formulation of One Care Plan, including comprehensive needs assessment and screening; and (LC) Execution of One Care Plan, encompassing shared care, anticipatory care, escalation management, care coordination, and strengthening of protective health factors. This structure enables the dashboard to link specific interventions (processes) to segment-level results (outcomes), supporting identification of where care delivery may require strengthening for particular population segments.

As noted in Section 3.1, initial implementation prioritised outcome indicators for which data were readily available, with process and output indicators planned for consolidation from operational systems in subsequent phases.

#### 3.2.1. Resident Distribution by Lifelong Segment

**Figure 3:**
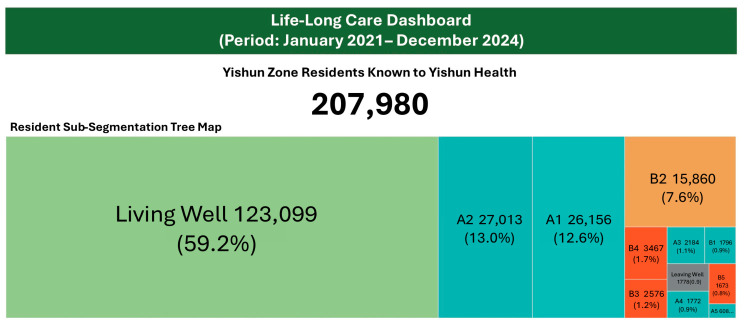
Lifelong Care Dashboard: population segment distribution for Yishun Zone residents known to Yishun Health (January 2021 to December 2024).

Over the dashboard period (January 2021 to December 2024), the dashboard tracked 207,980 Yishun Zone residents known to Yishun Health (Figure 3). This population-level visibility across all sub-segments, previously unavailable, enabled system stewards to better understand the composition of their catchment population and plan services accordingly.

#### 3.2.2. Longitudinal Health Outcomes by Lifelong Segment

**Figure 4:**
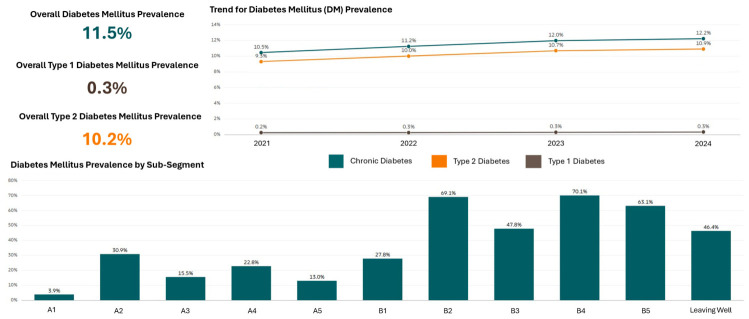
Longitudinal trends in chronic disease prevalence among Yishun Zone residents (2021–2024).

Unlike the cross-sectional validation reported in the Systemic Population Segmentation paper [27], the dashboard enabled the longitudinal monitoring of population health trajectories. Figure 4 illustrates trends in chronic disease prevalence (diabetes, hypertension, hyperlipidaemia) from 2021 to 2024, disaggregated by sub-segment. These longitudinal trends informed preventive health programme planning by identifying segments with rising disease burden.

#### 3.2.3. Quality Indicators by Lifelong Segment

**Figure 5:**
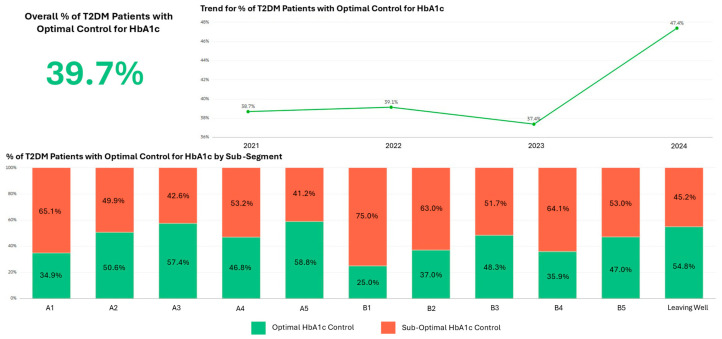
Optimal HbA1c control among residents with Type 2 diabetes by sub-segment.

The dashboard enabled a sub-segment-level quality monitoring that was not previously available. Figure 5 shows optimal HbA1c control rates among residents with Type 2 diabetes, revealing trends and substantial variation across sub-segments. Notably, sub-segments with psychosocial complexity did not necessarily demonstrate a worse quality indicator performance than their counterparts without psychosocial issues, a pattern consistent with the possibility that appropriately supported care may help mitigate some effects of complexity.

#### 3.2.4. Intervention Process Indicators

**Figure 6:**
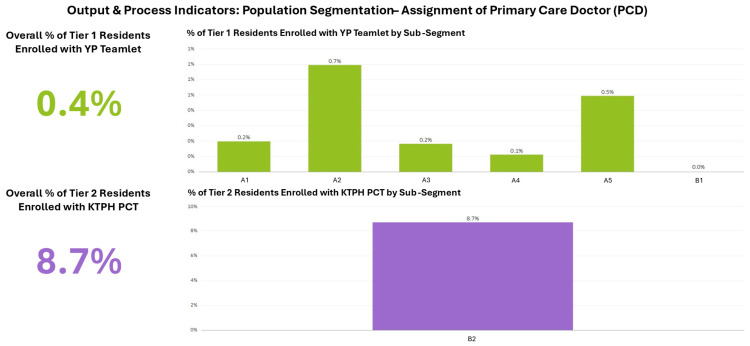
Primary Coordinating Doctor assignment rates by population tier and sub-segment.

**Figure 7:**
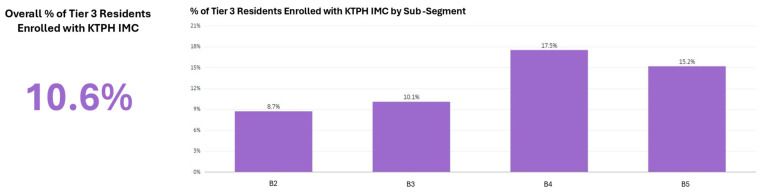
Integrated Medical Clinic enrolment rates for Tier 3 residents by sub-segment.

Beyond outcome measurement, the dashboard incorporated indicators tracking key interventions in the One Care Plan journey. Figure 6 shows Primary Coordinating Doctor (PCD) assignment rates by population tier and sub-segment, while Figure 7 shows Integrated Medical Clinic (IMC) enrolment trends for Tier 3 residents requiring hospital-based chronic disease management. These process indicators represent examples of the intervention-tracking capabilities specified in the indicator framework (Appendix A), creating a foundation for future analysis of how care programme coverage relates to segment-level outcomes. As data infrastructure develops, additional process indicators including screening rates, care plan completion, and vaccination coverage will expand this intervention monitoring capability.

#### 3.2.5. Cost by Lifelong Segment and Care Setting

**Figure 8:**
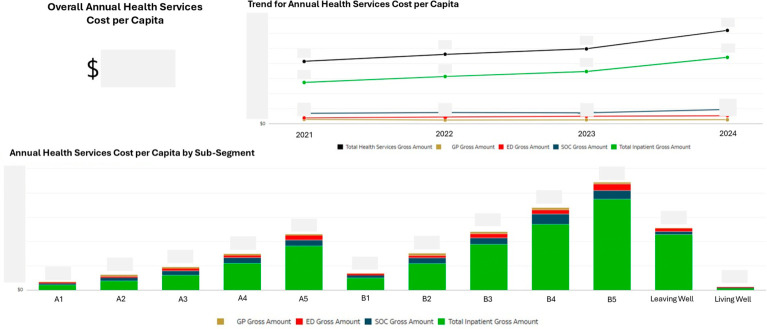
Annual health services cost per capita by care setting and sub-segment (actual cost figures are masked to adhere to relevant data governance policies).

While the Systemic Population Segmentation paper reported total annual healthcare costs by sub-segment [27], the Lifelong Care Dashboard provided decomposition by care setting, revealing where costs accumulated within the integrated regional population health system (Figure 8). Cost profiles varied across sub-segments, with inpatient costs dominating for high-complexity segments while lower-complexity segments showed more even distribution across care settings. This decomposition informed discussions about care setting appropriateness and the potential for upstream investment in community-based care.

### 3.3. Episodic Care Dashboard

The Episodic Care Dashboard is designed to provide continuous monitoring of health and outcomes for patients during acute care episodes when they are escalated from Lifelong Care to Episodic Care. It offers insight into acute and transitional care processes and outcomes, stratified by presenting acuity and Lifelong Care segment status using the Systemic Health System Population Segmentation Model.

The dashboard provides filtering by Episodic Care segment, admission type (emergency, elective, day surgery), discharge period, subspecialty, diagnosis-related group, patient class, ward type, and zone status. The zone filter enables comparison between Yishun Zone residents, who benefit from continuity with the integrated care system, and non-residents presenting without prior relationship to the regional population health system.

Indicators are organised into two complementary domains (Appendix B). Outcome indicators follow the Patient Value Compass framework: functional status (Modified Barthel Index, EQ-5D-5L, mental health improvement), clinical outcomes (mortality, readmissions, length of stay, hospital-acquired complications), experience (patient satisfaction), and cost to patients (episodic cost, days away from meaningful work). Process and output indicators are structured around the Episodic Care Plan journey in four phases: (EA) Initiation of Episodic Care; (EB) Formulation of Episodic Care Plan; (EC) Execution of Episodic Care Plan; and (ED) Transition to Lifelong Care Plan. This structure enables the dashboard to trace patient journeys through acute care and link care processes to segment-specific outcomes, informing targeted quality improvement efforts.

#### 3.3.1. Case Distribution by Admission Type and Episodic Segment

**Figure 9:**
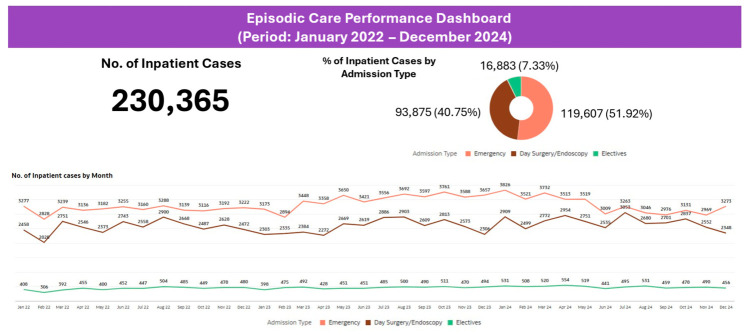
Episodic Care Dashboard: inpatient case distribution by admission type (January 2022 to December 2024).

**Figure 10:**
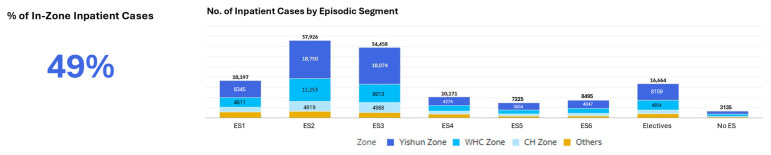
Episodic Care Dashboard: inpatient case distribution by episodic care segment (January 2022 to December 2024).

Over the three-year period (January 2022 to December 2024), the dashboard tracked 230,365 inpatient cases. This scope, encompassing emergency, elective, and day surgery cases (Figure 9), enabled comprehensive pattern identification. Approximately 49% of inpatient cases were Yishun Zone residents (Figure 10).

#### 3.3.2. Clinical Outcomes by Episodic Segment

**Figure 11:**
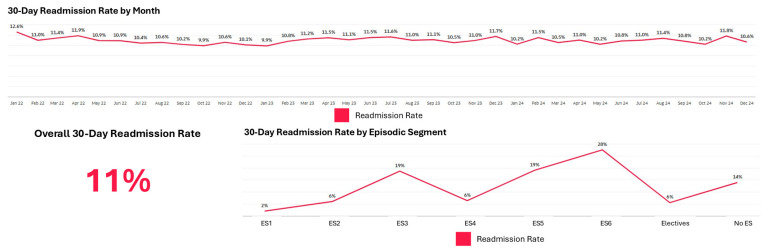
Thirty-day readmission rate by episodic care segment.

The dashboard enabled segment-level clinical outcome monitoring. Figure 11 shows 30-day readmission rate trends by episodic segment, revealing substantial variation consistent with patterns identified in the Systemic Population Segmentation paper [27]. Segments with psychosocial complexity (ES3, ES5, ES6) demonstrated higher readmission rate trends than segments with similar medical acuity but without psychosocial issues, reinforcing the importance of addressing psychosocial needs during acute care episodes.

**Figure 12:**
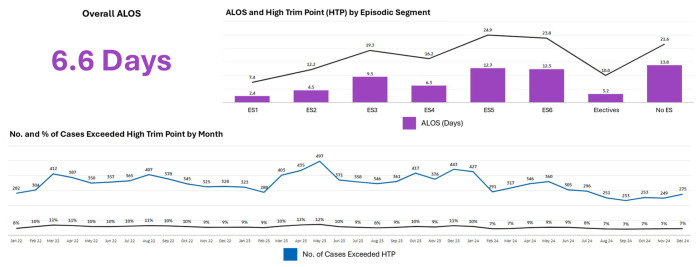
Average length of stay and High Trim Point by episodic care segment, with temporal trends.

Figure 12 shows average length of stay and High Trim Point thresholds by segment. The percentage of cases exceeding High Trim Point thresholds showed improving trends over the measurement period, a pattern that coincided temporally with the introduction of segment-based length-of-stay management initiatives.

Other important experience outcomes (patient satisfaction by segment) and functional outcomes (Modified Barthel Index improvement), as well as key process and output indicators for the Episodic Care Plan journey, while specified in Appendix B, have not yet been consolidated into this dashboard from our clinical quality, patient experience, and operational hospital data tracking systems. These are planned for subsequent phases of development, as described in Section 3.1.

#### 3.3.3. Cost by Episodic Segment

**Figure 13:**
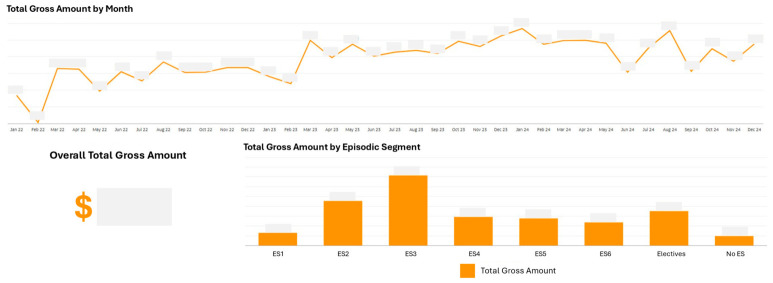
Total inpatient costs by episodic care segment (2022–2024) (actual cost figures are masked to adhere to relevant data governance policies).

A key capability enabled by the dashboard was aggregate cost trend visibility by segment (Figure 13). Consistent with patterns identified in the Systemic Population Segmentation paper [27], segments with psychosocial complexity accounted for disproportionate costs. The dashboard’s aggregate and trend view revealed the magnitude of this disparity at system level, with segment-level cost concentration informing resource allocation discussions.

**Figure 14:**
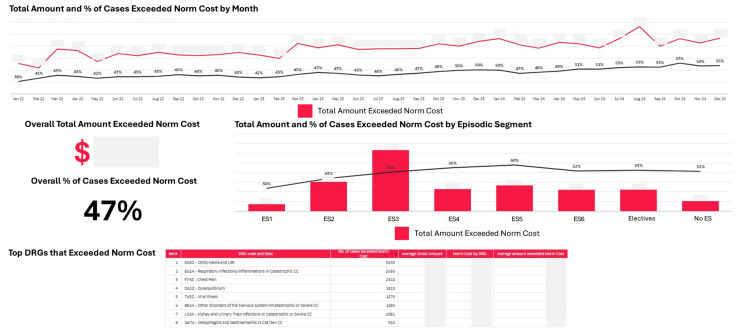
Norm cost analysis showing excess costs by episodic care segment (actual cost figures are masked to adhere to relevant data governance policies).

The dashboard tracked cases exceeding normative cost thresholds, enabling the identification of efficiency improvement opportunities (Figure 14). The analysis revealed variation across segments in both the proportion of cases exceeding norm costs and the magnitude of excess costs, providing actionable intelligence for targeted cost management initiatives.

### 3.4. Systemic Insights Enabled by Dashboard Architecture Based on Systemic Population Segmentation and Systemic Model of Care

The parallel structure of the Lifelong Care and Episodic Care dashboards, built upon the same population segmentation model, enables insights that span the care continuum. Because segments are defined consistently across both dashboards, patterns observed in lifelong care can be examined alongside patterns in episodic care, revealing systemic relationships that siloed measurement approaches would obscure.

#### 3.4.1. Psychosocial Complexity as a Cross-System Cost Determinant

The dashboards revealed psychosocial complexity as consistently associated with higher costs across both care systems. In the Lifelong Care Dashboard, sub-segments with psychosocial issues demonstrated higher per capita costs than counterparts with similar medical complexity but without psychosocial issues (Figure 8, Section 3.2). In the Episodic Care Dashboard, ES3 (Low Acuity with Psychosocial Issues) accounted for the largest share of total inpatient costs despite not being the highest acuity segment and similarly dominated excess costs above normative thresholds (Figure 13 and Figure 14, Section 3.3). This convergent pattern across both dashboards is consistent with the findings of the Systemic Population Segmentation paper [27] and provides an impetus for us to establish service quality improvements and redesigns to improve how we address psychosocial complexity.

#### 3.4.2. Informing Service Redesign Decisions

Dashboard visualisations revealing performance variation by segment prompted clinical leadership to evaluate existing service delivery models. For example, the Integrated Medical Clinic team, reviewing segment-level utilisation and cost patterns, initiated discussions about whether lower-intensity segments could transition to polyclinic-based care with our integrated care zone, reserving hospital-based specialist care for higher-complexity segments. The Episodic Care Dashboard’s segment-level length of stay and readmission data similarly informed discussions about ward-based care processes and resource allocation for psychosocially complex segments.

#### 3.4.3. Future Analytical Capabilities Through Common Segmentation

The indicator framework (Appendix A and Appendix B) specifies relationships that, when fully operationalised, will enable more sophisticated systemic analysis. The Lifelong Care Dashboard tracks chronic disease quality indicators by sub-segment (Figure 4, Section 3.2), while the Episodic Care Dashboard tracks clinical outcomes including readmission rates by segment (Figure 11, Section 3.3). Linking these indicators would enable analysis of whether residents with suboptimal chronic disease control experience higher rates of acute utilisation. Similarly, linking intervention process indicators such as Primary Coordinating Doctor assignment (Figure 6, Section 3.2) to episodic care outcomes would enable evaluation of whether residents enrolled in structured care programmes experience different acute care patterns. These cross-dashboard linkages represent future analytical capabilities that the common segmentation architecture is designed to support.

Taken together, these observations illustrate how the dashboard architecture enabled insights and discussions that would not have been possible without segment-level measurement visibility across both lifelong and episodic care systems. The consistent finding that psychosocial complexity is associated with higher costs and poorer outcomes across both dashboards, combined with the ability to link lifelong care status to episodic care patterns, illustrates the potential value of systemic measurement architecture for health system governance and quality improvement. The implications of these findings for population health systems transformation, including how such measurement approaches might be adapted by other health systems, are considered in the following section.

## 4. Discussion

### 4.1. Systemic Measurement Enables Systemic Accountability and Quality Improvement

The primary contribution of the Systemic Health System Measurement Framework lies in its demonstration that measurement architecture can be explicitly designed to enable systemic accountability to be applied more coherently across population segments and then at whole-health-system levels. Conventional measurement approaches often struggle to clarify who is accountable for what outcomes, particularly in integrated care environments where multiple provider organisations collaborate to serve shared populations [9]. The SHSMF addresses this challenge by aligning measurement architecture with the purposes, boundaries, and accountability structures defined through the Unified Care Model and operationalised through the Systemic Health System Population Segmentation Model.

This paper completes a conceptual systemic health systems design and transformation trilogy addressing three interdependent questions for people-centred and value-based health system transformation, i.e., for whom will care be integrated (systemic population segmentation), what interventions will be delivered (segment-specific care models based on the Unified Care Model), and how will we know whether integration is succeeding (both systemic and systematic measurement). The SHSMF operationalises the first two components into ongoing measurement visibility, enabling the continuous feedback essential for adaptive population health system performance management and governance.

The case study findings are consistent with the view that measurement architecture explicitly designed around needs-based population segments may reveal patterns that may not be apparent through conventional provider-centric or disease-based measurement. The Lifelong Care Dashboard revealed substantial cost variation across sub-segments, with the highest-complexity residents incurring costs many times greater than Living Well residents, a pattern that appears to be associated with higher emergency inpatient utilisation rather than planned care.

The parallel structure of the Lifelong Care and Episodic Care Dashboards provided ongoing performance management data consistent with a pattern identified in our segmentation validation work [27]: psychosocial complexity, rather than medical acuity alone, appears to be associated with higher healthcare costs across both care systems. In the Episodic Care Dashboard, ES3 (Low Acuity with Psychosocial Issues) accounted for the largest share of total inpatient costs and excess costs beyond normative thresholds, despite representing patients with lower medical acuity than other emergency segments. This convergent finding across both dashboards has implications for resource allocation and services redesign and quality improvements, and is consistent with the view that investments in mental health support, social work capacity, and community-based psychosocial interventions may be associated with greater system-level value alongside continued investment in high-acuity medical services.

A notable finding from the Lifelong Care Dashboard was that sub-segments with psychosocial complexity did not necessarily demonstrate a worse quality indicator performance. For residents with Type 2 diabetes, some psychosocially complex sub-segments achieved comparable or better glycaemic control than medically complex sub-segments without psychosocial issues, a finding consistent with the possibility that appropriately designed and resourced care may help mitigate the effects of complexity. This is consistent with the view that psychosocial complexity does not inevitably lead to poorer outcomes, and the dashboard’s ability to surface such variation prompted clinical leadership to evaluate whether existing service delivery models were appropriately designed for all population segments. These discussions represent a shift from uniform service models toward segment-informed service design.

These examples of systemic insights illustrate how the SHSMF addresses several of the measurement gaps identified in Section 1.2 and Table 1, extending and operationalising existing frameworks within a unified, segment-based measurement architecture. By organising structure, process and outcome indicators around needs-based population segments and cascading them across system levels, the SHSMF extends Donabedian’s evaluative logic [11] from individual services to whole-system performance. By integrating measurement across conditions within each segment rather than within single-condition cycles, it complements the condition-specific depth of ICHOM outcome sets [14], clinical registries [15], and Porter’s value-based healthcare framework [12,13] with a population-level perspective. The SHSMF more explicitly operationalises the Triple and Quadruple Aim goal domains [19,20] as the indicator framework for Lifelong Care measurement and adapts the Patient Value Compass [28] for Episodic Care segments, translating these widely adopted visions into segment-level measurement architecture. It offers operational specificity at the regional health system level that complements and builds upon the OECD Healthcare Quality Indicators Framework [16] and the WHO Health System Performance Assessment framework’s [18] national-level orientation, providing a more specific measurement architecture through which Learning Health System [21] improvement cycles can operate across population segments and governance tiers of performance management. While these relationships warrant further empirical examination, the case study findings are consistent with the view that a systemic measurement architecture organised around needs-based population segments may complement and extend the contributions of existing frameworks.

Our experience also demonstrated that systemic measurement architecture creates a foundation for strategic planning. The mutually exclusive and collectively exhaustive segmentation model enabled population-based resource allocation discussions, while dashboard visualisations supported coalition-building within our organisation’s numerous departments and also with partner institutions, leading to better service planning conversations and data sharing agreements. This illustrates how measurement architecture can serve as an enabler of health system transformation rather than merely as a reporting function.

### 4.2. Framework Adaptation for Other Health Systems

While developed within a Singaporean regional population health system, the SHSMF’s design principles offer transferable methodology for health systems globally seeking to implement systematic measurement for population health transformation.

#### 4.2.1. Start with Available Data, Organised Differently

Patient-centric, needs-based measurement represents an aspiration that must often be pursued within the constraints of existing provider-centric data structures. Most health systems possess substantial administrative and clinical data organised around provider institutions, clinical departments, or disease programmes. The SHSMF case study at Yishun Health indicates that reorganising existing data around population segments, even when the underlying data sources remain provider-centric, appears to be linked to the identification of patterns not readily visible in conventional reporting. Health systems need not wait for a perfect data infrastructure before implementing segment-based measurement; rather, they can begin with available data while progressively enhancing data capture for psychosocial determinants and resident-reported outcomes [31].

#### 4.2.2. Adopt Cascade Architecture Appropriate to Governance Structures

Our conceptual three-level SHSMF cascade (macrosystem, mesosystem, and microsystem) provides a generalisable systemic structure for systematic measurement in value-based health systems, but the specific configuration should reflect local governance arrangements [17]. Health systems organised as integrated delivery networks may implement the cascade within a single organisation; those comprising collaborating independent providers may require inter-organisational governance forums at each level. The essential principle is systemic design and alignment: indicators at each level should aggregate appropriately and serve the accountability and improvement purposes of stakeholders at that level, before aggregating into a whole.

#### 4.2.3. Integrate Lifelong and Episodic Care Measurement

All population health systems must grapple with allocating resources between longitudinal health maintenance and acute crisis response. The SHSMF’s integration of Lifelong Care and Episodic Care measurement, which links each resident’s episodic events to their lifelong care segment membership based on the Unified Care Model, enables analysis of how upstream factors may influence downstream outcomes and how acute events may affect long-term trajectories. This bidirectional linkage, while technically demanding, has the potential to provide strategic intelligence unavailable from siloed measurement systems.

#### 4.2.4. Establish Governance Forums Before Perfecting Measurement

Measurement generates value only when it informs decisions and actions. Population health systems should establish governance forums at each cascade level (strategic committees reviewing population-level outcomes, programme boards reviewing segment-specific performance, care teams reviewing operational indicators) concurrent with dashboard development. These forums create demand for measurement, surface requirements for indicator refinement, and build cultures of data-informed improvement, including resolving the needed tensions to better clarify roles and improve performance management accountability as health systems become more integrated and more systemic. Waiting until measurement systems are perfected before establishing governance risks creating technically sophisticated dashboards that remain unused.

### 4.3. Practical Lessons from Implementation

Several implementation lessons emerged with potential relevance for other health systems.

#### 4.3.1. Create Visualisations Before Securing Data Access

Dashboard mock-ups enabled a demonstration of the value proposition to stakeholders controlling data and resources, transforming abstract measurement discussions into concrete visualisations. When engaging the polyclinic institution for data sharing, mock-ups using hospital data for segments typically managed by polyclinics generated enthusiasm for collaboration before formal approvals were secured. This approach appeared to facilitate coalition building that might otherwise have stalled in protracted data governance negotiations. We recommend that health systems develop compelling visualisations of intended dashboards early in the process, using available data or synthetic examples, to build stakeholder commitment.

#### 4.3.2. Engage Willing Participants First

Not all departments or partner organisations engage at similar pace. Rather than mandating organisation-wide participation, pragmatic prioritisation based on demonstrated readiness enabled iterative refinement with early adopters before broader deployment. This approach aligns with complexity-informed change management: collaborating with willing participants creates demonstration effects that can shift organisational culture more effectively than mandated compliance [32]. Health systems should identify clinical leaders and departments showing enthusiasm for population health measurement and concentrate initial implementation efforts there.

#### 4.3.3. Design for Dual Purposes During Transition

Clinical stakeholders emphasised that dashboards needed to balance future-oriented population health indicators with current operational metrics. Designing dashboards to serve both purposes, displaying segment-based population outcomes alongside familiar institutional performance indicators, increased complexity but proved essential for securing support during the transition from provider-centric to population-centric measurement paradigms. Health systems should anticipate this dual requirement and design measurement systems that can demonstrate value within existing accountability frameworks while building toward transformed approaches.

#### 4.3.4. Accept Incremental Progress and Expect Continuous Maturation

Full realisation of needs-based measurement requires data linkage across providers, care settings, and social services, a goal that may take years to achieve [31]. Our implementation proceeded with available hospital data while working in parallel to establish data sharing with polyclinics and community providers. Health systems should not delay implementation awaiting perfect data; rather, they should begin with available sources and demonstrate value. The insights presented in this case study reflect ongoing maturation, with data quality and indicator coverage continuing to improve as process indicators are consolidated, data sharing agreements expand, and data capture workflows become embedded in clinical practice. Initial implementations establish the architecture and demonstrate value; subsequent cycles progressively enhance analytical capability.

### 4.4. Implications for Policy and Practice

The SHSMF development and implementation, especially in the context of our systemic health systems design and transformation trilogy, has several implications for health system stewards and policy makers seeking to accelerate transformation toward value-based population health models.

#### 4.4.1. Design Measurement Systemically from Transformation Outset

Beginning with systemic population segmentation, then developing segment-specific care interventions, and finally implementing aligned systemic measurement, ensures coherence between what health systems aim to achieve, how we organise to achieve it, and how we know whether we are succeeding. Measurement architecture should be designed as a foundational systemic enabler rather than a retrospective addition.

#### 4.4.2. Make Psychosocial Determinants Visible with Equal Rigour

Our descriptive findings are consistent with the evidence that psychosocial factors are often associated with higher healthcare utilisation and poorer outcomes than medical complexity alone [33,34]. However, many conventional measurement frameworks treat psychosocial factors as secondary considerations. Health system stewards should prioritise indicator sets capturing psychosocial determinants, advocate for policies enabling the linkage of healthcare and social service data, and ensure resource allocation explicitly considers psychosocial complexity alongside medical needs.

#### 4.4.3. Operationalise Accountability at the Population Segment Level

In integrated care environments involving multiple providers, ambiguity about accountability undermines transformation efforts. Segment-specific measurement, where care teams are designated as accountable for outcomes and costs for specific population segments, clarifies accountability in ways that institutional or disease-based measurement cannot [35]. Policy makers should develop financing and governance mechanisms supporting explicit segment-level accountability.

#### 4.4.4. Position Measurement for Learning, Not Merely Reporting

The SHSMF’s emphasis on timely dashboards, regular governance reviews, and continuous improvement cycles reflects a Learning Health System orientation where measurement generates rapid feedback, enabling adaptation [21]. System stewards should position measurement as strategic intelligence for proactive management rather than a compliance burden, investing in user-friendly dashboards, training for data interpretation, and cultures where performance variation triggers collaborative problem-solving.

## 5. Limitations

Several limitations of the SHSMF and its implementation warrant acknowledgment, providing direction for future refinement and research. Many of these limitations relate to the underlying data infrastructure and are shared with the population segmentation models described in the Systemic Population Segmentation paper of this trilogy [27].

### 5.1. Data Coverage and Quality Constraints

At this stage of development, our implementation of the SHSMF into Lifelong Care and Episodic Care dashboards still predominantly relies on administrative data from electronic medical record and hospital information systems, e.g., for population segmentation. While this enables scalable, automated measurement across large populations, important data gaps constrain measurement comprehensiveness.

Psychosocial determinants are captured through proxy indicators (medical social worker visits, public rental housing status as a marker of low socioeconomic status, and ICD-10 diagnosis codes for mental and behavioural disorders in the F00-99 categories) rather than comprehensive assessments of residents’ social circumstances, mental health status, support networks, health literacy, and health behaviours. These proxies capture only formally diagnosed conditions and documented social needs, likely underestimating the true prevalence of psychosocial complexity among residents who have not accessed specific services or received formal diagnoses.

Functional status measures can begin to be taken from clinical and operational transactional systems, but the data remains incomplete in these source systems. Our dashboards rely on Clinical Frailty Scale scores and inpatient assessments but lack systematic community-based measurement and institutional transaction systems collection. Caregiver availability and informal support networks, critical determinants of care needs, are not yet systematically captured.

These data limitations mean that some framework indicators rely on administrative proxies that may misclassify residents’ true needs. Additionally, our dashboards currently capture data primarily from Yishun Health institutions, with polyclinic data obtained through negotiated sharing agreements. Future refinement should prioritise the systematic collection of resident-reported assessments and functional status measures across care settings, comprehensive social determinants data, and extended data capture from community-based providers, including independent primary care clinics and social service organisations.

Finally, the current implementation focused primarily on macrosystem and mesosystem measurement. Microsystem-level dashboards enabling frontline care teams to monitor operational performance remained in early development. Extending the architecture to support microsystem-level feedback represents an important area for future enhancement, as frontline engagement is essential for translating measurement insights into care delivery improvements.

### 5.2. Outcome Attribution in Distributed Care

The findings that the SHSMF architecture makes visible do not aim to establish causal relationships or test statistical hypotheses. While the SHSMF clarifies accountability by assigning care teams responsibility for specific population segments, establishing the causal attribution of outcomes to specific interventions remains challenging in distributed care environments, where residents receive care from multiple providers. When a resident’s health status improves or utilisation decreases, isolating the relative contributions of their Primary Coordinating Doctor, specialist consultations, hospital-based interventions, and community support services is methodologically difficult. Similarly, when episodic care outcomes deteriorate, distinguishing inadequate care delivery from changes in social circumstances or natural disease progression poses challenges that segment-level measurement alone cannot resolve.

The framework addresses this through collective accountability, where care teams are accountable for average outcomes for their designated segments rather than precise attribution at individual level. While this pragmatic approach enables action and avoids analysis paralysis, it may obscure within-segment variation and limit the ability to identify which specific intervention components are most effective. Future services research with specific hypothesis testing will be needed, as the dashboard-level analyses in this paper were meant primarily for performance management and quality improvement and were not subject to inferential statistical testing.

### 5.3. Contextual Dependencies and Generalisability

The SHSMF dashboards implementation was developed within a specific regional population health system serving approximately 320,000 residents in an urban setting in Singapore. Its generalisability to other contexts remains to be empirically tested.

Several contextual features enabled implementation that may not be present elsewhere. Singapore’s integrated public healthcare system, with shared patient identifiers enabling data linkage across institutions, provided a foundational data infrastructure. The reorganisation of Yishun Health as an integrated care organisation with explicit population health stewardship responsibilities created governance structures supporting segment-level accountability. National policy alignment through the Three Beyonds initiative and Healthier SG programme provided strategic impetus for population health measurement.

Health systems in different contexts would face varying implementation challenges. Those lacking integrated data repositories or interoperable electronic record systems would encounter significant barriers to full framework implementation, though they might adapt the conceptual approach using available data sources. Systems without clear population denominators, which may be common in some insurance-based or more fragmented provider markets, would need to establish attributed populations before implementing segment-level measurement. Those operating under fundamentally different financing mechanisms, such as pure fee-for-service environments without value-based payment incentives, may lack the organisational motivation for population health measurement.

Despite these contextual dependencies, the framework’s core systemic design principles offer methodological guidance that is applicable across diverse settings, as discussed in Section 4.2. Adaptation would require tailoring segments to local disease patterns and social determinants, configuring cascade architecture to local governance arrangements, and working within the available data infrastructure while progressively enhancing data capture.

## 6. Conclusions

This paper presented the Systemic Health System Measurement Framework (SHSMF), completing a systemic health systems design and transformation conceptual trilogy with the Unified Care Model and Systemic Health System Population Segmentation Model. Together, these frameworks address three interdependent questions for health system transformation: for whom care will be integrated, what interventions will be delivered, and how will success be measured?

The SHSMF organises measurement around needs-based population segments, integrates lifelong and episodic care measurement, and cascades indicators across system levels to enable accountability at each governance tier. The case study at Yishun Health (2022–2024) illustrated that this architecture reveals patterns not readily visible through conventional approaches. Notably, psychosocial complexity is consistently associated with higher costs across both dashboards, with segments characterised by psychosocial issues accounting for disproportionate costs despite lower medical acuity.

For health systems seeking to implement value-based population health models, the SHSMF offers transferable principles: reorganise available data around population segments, align cascade architecture with governance structures, integrate longitudinal and episodic measurement, and establish governance forums concurrent with dashboard development. Systemic measurement, when aligned with systemic care model design and systemic population segmentation, enables systemic accountability for whole-population outcomes.

## Figures and Tables

**Table 1:** Common health system measurement frameworks and their scope for whole-system measurement.

Framework/Approach	Measurement Focus	Key Contribution	Scope Considerations for Whole-System Measurement
Donabedian’s Structure–Process–Outcome [11]	Quality of health services at the clinical and institutional levels	Established foundational principles linking care structures and processes to outcomes; remains the dominant framework for health services quality evaluation	Designed primarily for assessing quality within specific clinical services or institutions; its application has not typically extended to measurement across whole populations with multiple system levels simultaneously
Porter’s Value-Based Health Care [12,13] and International Consortium for Health Outcomes Measurement (ICHOM) Standardised Outcome Sets [14]; Clinical Registries [15]	Patient outcomes relative to costs across full cycles of care for specific medical conditions	Shifted focus from volume-based to value-based measurement; defined value as outcomes that matter to patients per unit cost; developed standardised outcomes measures for specific medical conditions and introduced Integrated Practice Units; clinical registries enabling cross-institutional benchmarking have demonstrated that adherence to standardised measurements is associated with improved patient outcomes	Condition-specific focus provides depth within individual diseases. Generally, does not integrate outcomes across conditions for populations with multiple concurrent needs. Needs more explicit guidance for implementation across provider organisations to integrate care or evaluate whole-system performance
OECD Healthcare Quality Framework [16]	Standardised quality indicators organised by dimensions of effectiveness, safety, and responsiveness across patient care needs (staying healthy, getting better, living with illness or disability, coping with end of life)	Developed a multi-dimensional framework for the international benchmarking of healthcare quality across OECD member countries; established indicator selection criteria (importance, scientific soundness, feasibility); organised indicators around quality dimensions and patient care needs across the life course	Primarily oriented toward international benchmarking at national level; organises indicators by quality dimensions and care needs rather than by population segments; offers less operational specificity for structuring measurement within regional or local population health systems
Nelson et al. Clinical Microsystems [17]	Quality improvement and care delivery across nested system levels: clinical microsystems (frontline care teams), mesosystems (clinical programmes linking microsystems) and macrosystems (overarching health organisations)	Established the structural principle that health systems are composed of nested levels where frontline clinical microsystems are the building blocks of system performance; articulated that improvement must operate across all levels simultaneously for organisation-wide results	Oriented primarily toward quality improvement within and between clinical units rather than toward population-level measurement architecture; does not organise measurement around needs-based population segments or integrate longitudinal and episodic care measurement within a unified framework
WHO Health System Performance Assessment [18]	System-level goals: health improvement, responsiveness, financial protection; four functions: stewardship, financing, service provision, and resource generation	Provides comprehensive conceptual guidance for assessing health system performance at the national level, linking system functions to overarching goals	Primarily oriented toward national-level assessment; offers less operational specificity for structuring measurement architectures that guide day-to-day decision-making within regional or local population health systems
Triple Aim/Quadruple Aim [19,20]	Goal domains: population health, patient experience, per capita cost; Quadruple Aim adds provider wellbeing	Widely adopted guiding vision articulating core goal domains for health system improvement	Articulates high-level goal domains but does not sufficiently specify measurement architecture for operationalising them within complex, multi-level health systems or across differentiated population segments
Learning Health System [21]	Continuous cycles of data generation, knowledge synthesis, and practice improvement	Envisions health systems that continuously learn and improve through the systematic use of data and evidence to inform clinical and operational decisions	Provides a compelling vision for continuous improvement but offers less guidance on the specific measurement architecture needed to operate across population segments and system levels simultaneously

## Data Availability

The data presented in this study are not publicly available due to privacy and confidentiality restrictions, as they contain patients’ medical and social information. The dataset was derived from Yishun Health’s Central Data Repository (CDR) containing administrative, clinical, and financial data. Requests to access the aggregated datasets may be considered by the corresponding author upon reasonable request and subject to approval from the relevant institutional data protection office and governance committees.

## Appendix A. Lifelong Care Dashboard Indicator List

**Table A1:** Outcome Indicators Framework—Quadruple Aims.

Domain	Code	Indicator
Population Health	L001	% Residents with Protective Health Factors: (a) Weight Status (Body Mass Index—BMI); (b) Smoking Status; (c) Physical Activity Status; (d) Immunisation Status
	L002	Chronic Disease Prevalence: (a) Diabetes Mellitus (DM); (b) Hypertension (HTN); (c) Hyperlipidaemia (HLD); (d) Dementia
	L003	Cancer Prevalence: (a) Breast; (b) Cervical; (c) Colorectal
	L004	Frailty prevalence
	L005	Health-Related Quality of Life (HRQoL)
	L006	Mental Well-Being
	L007	Age-Adjusted per Capita Bed Days in Hospital
Experience	L008	Residents’ Experience of Health
Costs	L009	Average Annual Health Services Utilisation per Capita: (a) General Practitioner (GP); (b) Polyclinic; (c) Specialist Outpatient Clinic (SOC); (d) Emergency Department (ED); (e) Episodes; (f) Inpatient Admissions, Including (i) Emergency and (ii) Elective
	L010	Annual Health Service Costs per Capita (Overall)
	L011	Institutionalisation Rates
	L012	Cost per Capita for Organisation to Deliver Services under One Care Plan (OCP): (a) Overall; (b) Subsidised Cost
Care Team Well-Being	L013	Employee Climate Survey Results (or Joy in Work Average Score)

**Table A2:** Output and Process Indicators Framework—Lifelong One Care Plan Journey.

Phase	Code	Indicator
(LA) Population Segmentation		
LA01. Assignment of Primary Coordinating Doctor (PCD)	P001	% Residents right sited (Overall)
	P001-a	% Tier 1 enrolled with Primary Coordinating Doctor (PCD) (by subsegment)
	P001-b	% Tier 2 enrolled with Primary Coordinating Doctor (PCD) (by subsegment)
	P001-c	% Tier 3 enrolled with Primary Coordinating Doctor (PCD) (by subsegment)
(LB) Formulation of One Care Plan		
LB01. Comprehensive Needs and Assets Assessment	P003-a	Screening rate (chronic disease): Diabetes Mellitus (DM)
	P003-b	Screening rate (chronic disease): Hypertension (HTN)
	P003-c	Screening rate (chronic disease): Hyperlipidaemia (HLD)
	P003-d	Screening rate (cancer): Breast
	P003-e	Screening rate (cancer): Cervical
	P003-f	Screening rate (cancer): Colorectal
	P003-g	% Residents with completed Advanced Care Plan (ACP) (ACP rate)
(LC) Execution of One Care Plan		
LC01. b. Shared Care	P008	Residents’ Activation
LC01. c. Anticipatory Care	P010-a	% Residents admitted to Khoo Teck Puat Hospital (KTPH) through elective flow
LC01. d. Escalation and Crisis Care	P011-a	Average monthly % of residents referred to Khoo Teck Puat Hospital (KTPH) Emergency Department (ED)
	P011-c	% Residents admitted to Khoo Teck Puat Hospital (KTPH) through emergency admission
LC02. Care Coordination	P012-a	AH and CH bed days in last 6 months of life
	P012-b	No. of visits at Yishun polyclinic per resident per year
	P012-c	Avoidable ED attendance rate
	P012-d	Ambulatory sensitive conditions (ACSC) hospitalisation rate
	P012-e	% Residents attending both Yishun Polyclinic and Yishun Health (YH) Medical SOC per year
	P012-f	Average no. of visits at Yishun Health (YH) Medical Specialist Outpatient Clinic (SOC) per resident per year
	P012-g	Average monthly referrals to Yishun Health (YH) Medical Specialist Outpatient Clinic (SOC) per 100 residents
	P012-h	Cost in Acute Hospital (AH) and Community Hospital (CH) in last 6 months of life
	P012-i	% Residents who died in-hospital
LC03. Strengthening of Modifiable Protective Health Factors	P013-a	Smoking cessation rate
	P013-b	% Obese residents (adults) with weight loss
	P013-c	% Obese residents (children) with weight loss
	P013-d	Children immunisation: Measles, Mumps, Rubella (MMR), and Diphtheria
	P013-e	Elderly immunisation: Influenza and Pneumococcal
	P013-f	Female immunisation: HPV
	P013-g	% Diabetes Mellitus (DM) patients with optimal control (HbA1c, Blood Pressure (BP), LDL)
	P013-h	% Diabetes Mellitus (DM) patients with appropriate screening done (eye, foot, renal)
	P013-i	% Hypertension (HTN) patients with optimal control
	P013-j	% Hyperlipidaemia (HLD) patients with optimal control

## Appendix B. Episodic Care Dashboard Indicator List

**Table A3:** Outcome Indicators Framework—Patient Value Compass.

Domain	Code	Indicator
Function	E001	Modified Barthel Index (MBI) score maintenance/improvement
	E002	EQ-5D-5L overall score
	E003	EQ-5D-5L no problems doing my usual activities Score
	E004	Improvement in: depression
	E005	Improvement in: cognition/dementia
	E006	Improvement in: anxiety
	E007	Improvement in: suicide risk
Clinical	E008	All-cause mortality
	E009	Inpatient all-cause mortality
	E010	Inpatient preventable deaths (Cat 3)
	E011	Case 30 day mortality from admission
	E012	≥1 all-cause emergency readmission
	E013	≥1 all-cause Emergency Department (ED) visits post discharge
	E014	Returns to Emergency Department (ED) post ED discharge
	E015	Episode Average Length of Stay (ALOS)
	E016	Pressure injuries
	E017	≥1 Serious Reportable Events (SRE)
	E018	≥1 Hospital-Acquired Infection (HAI)
	E019	≥1 Peripheral intravenous device-related phlebitis
	E020	≥1 medication error (Cat C to I)
	E021	Well-controlled pain (Inpatient)
	E022	Improvement in EQ-5D-5L score related to level of pain/discomfort
Experience	E023	Median Episodic Score in the Patient Experience Survey for: satisfaction with treatment received
	E024	Satisfaction with care teams’ communication
	E025	Satisfaction with patient and family empowerment, and care transition
	E026	Overall satisfaction with Yishun Health Campus (YHC) across all episodic sites
Cost to Patients	E027	Median episodic cost
	E028	Median episodic no. of days away from meaningful work

**Table A4:** Output and Process Indicators Framework—Episodic Care Plan Journey.

Phase	Code	Indicator
(EA) Initiation of Episodic Care	E029	P2 Accident and Emergency (A&E) consultation wait time
	E030	Emergency Department (ED) admission wait time
	E031	Subsidised Specialised Outpatient Clinic (SOC) lead time
(EB) Formulation of Episodic Care Plan	E032	Subsidised Specialised Outpatient Clinic (SOC) patients reviewed by specialists
	E033	Inpatients reviewed by specialists
	E034	% Medical assessments performed within 4 h of inpatient admission at Khoo Teck Puat Hospital (KTPH)
	E035	Computed Tomography (CT) scan turnaround time
	E036	X-ray turnaround time
	E037	Rate of urgent Magnetic Resonance Imaging (MRI) turnaround time within 24 h of order
	E038	Lab turnaround time
	E039	Blue letter turnaround time
	E040	Care plan documentation
	E041	Discharge plan documentation
	E042	Acute Hospital (AH)–Community Hospital (CH) transfer rate
	E043	Wait time for admission to Community Hospital (CH)
	E044	% Khoo Teck Puat Hospital (KTPH) inpatients discharged before 1200 h
(EC) Execution of Episodic Care Plan	E045	Unscheduled returns to operating rooms
	E046	Unscheduled returns to Intensive Care Unit (ICU)/High Dependency (HD)
	E047	Patient falls
	E048	Hand hygiene compliance
	E049	Critical results communication and acknowledgement
	E050	Patient identification errors
	E051	Turnaround time from referral to assessment (physiotherapist/occupational therapist/speech therapist/medical social worker)
	E052	Turnaround time for surgery
	E053	Rate of Subsidised patients with Table of Surgical Procedure (TOSP) ≥5 operated by a specialist
	E054	Surgical safety checklist compliance
	E055	Pre-procedural safety protocol in Interventional Radiology (IR) compliance
(ED) Transition to Lifelong Care Plan	-	In development
